# A cognitive behavioral approach allows improving brace wearing compliance: an observational controlled retrospective study with thermobrace

**DOI:** 10.1186/1748-7161-9-S1-O79

**Published:** 2014-12-04

**Authors:** Alessandra Negrini, Sabrina Donzelli, Monia Lusini, Salvatore Minnella, Fabio Zaina, Stefano Negrini

**Affiliations:** 1 ISICO - Italian Scientific Spine Institute, Milan, Italy; 2 University of Brescia - IRCCS Don Gnocchi, Brescia, Italy

## Background

Results of brace treatment of Idiopathic Scoliosis are related to brace wearing.

## Aim

To verify if Cognitive Behavioural Approach (CBA) dispensed during Physiotherapic Scoliosis Specific Exercises (PSSE) sessions increases brace wearing.

## Design

Retrospective controlled cohort study nested into a clinical database.

## Methods

Setting. Outpatient tertiary referral clinic.

Population. Out of 778 patients, 246 fulfilled the inclusion criteria: Idiopathic Scoliosis; first brace prescription; regular use of Thermobrace heat sensor; two evaluations after bracing; age ³6; European Risser 0-3.

Evaluations. T0 (start of bracing), T1 (4 months), T2 (10 months).

Measurements. Brace wearing compliance (BWC) T0 to T1 (T0-T1) and T1 to T2 (T1-T2).

Treatment. CBA adjunctive sessions dispensed during PSSE (CBA-PSSE), after the standard CBA provided to all patients including: at prescription, 20’ by Medical Doctor (MD) and 30-45’ by Physiotherapist; at brace check, 10-15’ by MD and Orthopaedic Technician; at T1, 10-15’ by MD.

Groups. According to CBA-PSSE in T0-T1 period: CBA1 (143 patients) ³2 sessions; Poor-Adherence (PA1, 52) 1 session; Control (CON1, 51) 0 sessions . Similarly, according to CBA-PSSE in T1-T2 period: CBA2 (97),PA2 (78), CON2 (71). Combinations among the 6 groups in the two periods were checked.

Statistics. ANOVA for group comparisons.

## Results

Patients were 13.03±1.11 years old. Brace hours prescription: 21.93±1.77 (T0-T1), 21.03±1.79 (T1-T2). BWC: 91.06±12.63% (T0-T1), 91.64±14.3% (T1-T2).

We found no differences among groups in brace prescription and BWC in T0-T1.

In T1-T2:

CBA1 had more brace prescription than CON1 (P<0.01);

CBA1 and CON1 improved BWC more than PA1 (P<0.005);CBA2 and CON2 improved BWC more than PA2; patients who were both in CBA1 and in CBA2, had more hours of brace prescription than PA2 and CON2, and improved brace compliance more than PA2 (P<0.05).

Overall, BWC differences among groups reached a maximum of 7%.

## Conclusion

CBA improves BWC: specifically, patients with high CBA-PSSE in both observed periods (CBA1+CBA2) had both the highest brace hours prescription and the highest BWC. Poor adherence to CBA-PSSE matched with poor adherence to brace wearing.

